# Cytotoxicity and antimicrobial action of selected phytochemicals against planktonic and sessile* Streptococcus mutans*

**DOI:** 10.7717/peerj.4872

**Published:** 2018-06-04

**Authors:** Marta Ribeiro, Joana Malheiro, Liliana Grenho, Maria Helena Fernandes, Manuel Simões

**Affiliations:** 1 LEPABE—Department of Chemical Engineering, Faculty of Engineering—University of Porto, Porto, Portugal; 2 Laboratory for Bone Metabolism and Regeneration, Faculty of Dental Medicine, University of Porto, Porto, Portugal; 3 REQUIMTE/LAQV, University of Porto, Porto, Portugal

**Keywords:** Biofilm control, Dental caries, Phytochemicals, Cytotoxicity, *Streptococcus mutans*

## Abstract

**Background:**

Dental caries remains the most prevalent and costly oral infectious disease worldwide, encouraging the search for new and more effective antimicrobials. Therefore, the aim of this work was to study the antimicrobial action of selected phytochemicals (eugenol, citronellol, sabinene hydrate, trans-cinnamaldehyde, terpineol and cinnamic acid) against *Streptococcus mutans* in planktonic and biofilm states as well as the cytotoxicity of these compounds.

**Methods:**

The antibacterial activity of the selected compounds was evaluated by the determination of the minimal bactericidal concentration. The resazurin assay was used to assess the metabolic activity of sessile *S. mutans*. The cytotoxicity was determined using a fibroblast cell line.

**Results:**

Among the tested phytochemicals, citronellol, cinnamic acid and trans-cinnamaldehyde were the most effective against both planktonic and sessile *S. mutans*, an effect apparently related to their hydrophobic character. Additionally, these three compounds did not compromise fibroblasts cell viability.

**Discussion:**

Citronellol, cinnamic acid and trans-cinnamaldehyde demonstrated significant antimicrobial activity and low cytotoxicity proposing their potential as a novel group of therapeutic compounds to control oral infectious diseases. Moreover, their effects are particularly relevant when benchmarked against eugenol, a phytochemical commonly used for prosthodontic applications in dentistry.

## Introduction

Oral diseases continue to be a major health problem worldwide. Dental biofilm formation can lead to the development of oral infectious diseases, such as caries, gingivitis and periodontitis (Chinsembu, 2016; Hwang, Klein & Koo, 2014). Dental caries is one of the most important global oral health problems, which is mainly associated with oral pathogens, particularly cariogenic *Streptococcus mutans* (Chinsembu, 2016; Gross et al., 2012; Hwang, Klein & Koo, 2014). *S. mutans* has the ability to metabolize several carbohydrates into organic acids that reduce the pH of dental plaque biofilm, causing the demineralization of tooth enamel and, consequently, leads to the initiation of dental caries. This bacterium is also a crucial contributor to the formation of a matrix of extracellular polymeric substances (EPS) on dental biofilms. Moreover, *S. mutans*-derived exopolysaccharides, mostly glucans, provide binding sites that promote accumulation of other microorganisms on the tooth surface and further establishment of cariogenic biofilms. The potential of *S. mutans* to survive under environmental stresses, such as acid conditions, high temperature and osmotic pressure, are other major virulence factors of this microorganism (Kawarai et al., 2016; Kwon et al., 2016; Liu & Yu, 2017; Zhang et al., 2016). Therefore, *S. mutans* should be a prime target for any therapeutic agent aimed at preventing dental caries.

Currently, the antibacterial agents used on the mouth, as mouth rinses or toothpastes, may inhibit the growth of *S. mutans* , preventing the development of dental caries. It is well-known that antibacterial mouth rinses are effective in decreasing tooth surface plaque (Quirynen et al., 2001). These mouth rises may contain fluorides, alcohols, detergents, and other synthetic antimicrobials compounds that include povidone iodine products, chlorhexidine and cetylpyridinium chloride. Some toothpastes also contain fluorides and other antimicrobials including triclosan and zinc citrate (Baehni & Takeuchi, 2003; Quirynen et al., 2001; Sheen, Eisenburger & Addy, 2003). However, there is an increasing pressure to substitute synthetic antimicrobials, which already gave rise to concerns regarding their toxicological and ecotoxicological properties (Dann & Hontela, 2011). In parallel, microorganisms will continue acquiring resistance to synthetic antimicrobial agents, which has encouraged the search for alternative products (Allaker & Douglas, 2009; Chuanchuen et al., 2001; Upadhyay et al., 2014).

Nowadays, natural antibacterial compounds in particular plant-derived compounds, are attracting attention to develop novel therapeutics against oral infectious diseases (Allaker & Douglas, 2009; Borges et al., 2016). Eugenol is a good example of natural compounds widely used in dental care as an antimicrobial, analgesic and anti-inflammatory, showing to be active against caries-related oral bacteria (Jadhav et al., 2004; Li et al., 2012; Xu et al., 2013). These plant-derived natural compounds, also referred as phytochemicals, are responsible for plant interactions with the environment. They are an attractive source of eco-friendly, relatively inexpensive and widely available new broad-spectrum antimicrobials with low levels of cutaneous cytotoxicity and environmental toxicity (Abreu et al., 2016; Borges et al., 2014a; Borges et al., 2016; Borges et al., 2017b; Dahiya & Purkayastha, 2012; Upadhyay et al., 2014). Furthermore, the multiple antimicrobial mechanisms of action of phytochemicals can prevent the emergence of resistant bacteria (Dahiya & Purkayastha, 2012; Upadhyay et al., 2014).

Essential oils (EOs) have been thoroughly explored in several studies, showing their broad-spectrum antimicrobial properties against both Gram-positive and Gram-negative bacteria (Bazargani & Rohloff, 2016; Prabuseenivasan, Jayakumar & Ignacimuthu, 2006; Sieniawska et al., 2013; Szczepanski & Lipski, 2014). These phytochemicals have also been reported to possess significant anti-inflammatory, antioxidant, anticancer, immune modulatory and regenerative activities (Bayala et al., 2014; Burt, 2004; De Cassia da Silveira e Sa, Andrade & De Sousa, 2013; Sadlon & Lamson, 2010; Woollard, Tatham & Barker, 2007). Despite the research progress on the antimicrobial activity of some EO components against oral bacteria, such as eugenol, many others remain largely unknown in the field of dentistry. In the present study, in order to provide further evidences on the antimicrobial potential of selected EO components, the antibacterial activity of citronellol, sabinene hydrate, trans-cinnamaldehyde, and terpineol were evaluated against *S. mutans* in both planktonic and sessile states, using eugenol as reference. The selection of phytochemicals was based on their promising effects in microbial growth control (Borges et al., 2017a; Lopez-Romero et al., 2015; Mith et al., 2014; Sharma et al., 2016; Szweda & Kot, 2017). Additionally, and based in a previous study (Malheiro et al., 2016), cinnamic acid, a phenolic acid, was also included in this study given its efficacy in the control of sessile bacteria, with activity similar to the benchmarked disinfectants, including peracetic acid, sodium hypochlorite and hydrogen peroxide. Furthermore, given the fact that the antimicrobial action of these compounds is known to be strictly correlated with their structure (Borges et al., 2017a), a drug-likeness evaluation based on the the chemical and molecular properties of these compounds was also carried out. Finally, the phytochemicals were evaluated for their cytotoxicity against a fibroblast cell line.

## Materials and Methods

### Bacterial strain and culture conditions

*S. mutans* DMS 20523 was used in all experiments. The bacterium was preserved at −80 °C in Tryptic Soy Broth (TSB, Oxoid, Basingstoke, UK) containing 30% (v/v) glycerol (Panreac, Barcelona, Spain). The bacterial cultures were grown overnight in TSB at 37 °C under 160 rpm of agitation before the experiments.

### Phytochemicals

Trans-cinnamaldehyde, sabinene hydrate, eugenol and terpineol (Table 1) were obtained from Sigma-Aldrich (Lisbon, Portugal); cinnamic acid was obtained from Merck (Lisbon, Portugal); citronellol was obtained from Acros Organics (Morris, NJ, USA). The structural and molecular properties of selected phytochemicals were determined with Molinspiration Calculation Software and Chemdraw (Malheiro et al., 2016). The phytochemicals were dissolved in dimethyl sulfoxide (DMSO, Sigma-Aldrich, St. Louis, MO, USA). Each compound was tested at various concentrations in the range of 1–25 mM in DMSO.

**Table 1:** Biological properties of the selected phytochemicals.

Phytochemical	Phytochemical class	Biological properties	References
Eugenol	Essential oil (phenol)	Antimicrobial, anti-inflammatory, antioxidant, anticancer, analgesic, sedative, spasmolytic.	Jadhav et al. (2004), Kamatou, Vermaak & Viljoen (2012), Lee et al. (2005) and Pengelly (2004)
Citronellol	Essential oil (alcohol)	Antimicrobial, anti-inflammatory, antioxidant, antiseptic, spasmolytic, tonifying.	Ćavar & Maksimović (2012), De Cassia da Silveira e Sa, Andrade & De Sousa (2013), Pengelly (2004) and Su et al. (2010)
Sabinene	Essential oil (hydrocarbon)	Antimicrobial, anti-inflammatory, anticancer, stimulant, decongestant.	Pengelly (2004) and Santoyo et al. (2006) and Sayyah et al. (2003)
Trans-cinnamaldehyde	Essential oil (aldehyde)	Antimicrobial, anti-inflammatory, antioxidant, sedative, spasmolytic, vasodilators.	Hill, Gomes & Taylor (2013), Pengelly (2004) and Tung et al. (2008)
Terpineol	Essential oil (alcohol)	Antimicrobial, anti-inflammatory, antiseptic, spasmolytic, tonifying.	Held, Schieberle & Somoza (2007) and Pengelly (2004)
Cinnamic acid	Phenolic acid	Antimicrobial, anti-inflammatory antioxidant, anticancer.	Liao et al. (2012), Song et al. (2013), Sova (2012) and Zhang et al. (2014)

### Minimum bactericidal concentration (MBC)

Minimum bactericidal concentration was determined according to Ferreira et al. (2011). Overnight cultures were centrifuged at 3,777 g for 15 min. Then, the supernatant of each bacterium was discarded and the cells were washed in NaCl solution (8.5 g/L, Merck, Germany) by resuspension and centrifugation at 3,777 g for 15 min. Cells were resuspended in fresh NaCl solution (8.5 g/L) and adjusted to a cell density of approximately 10^7^ cells/mL. Subsequently, 190 µL of the adjusted bacterial suspension were added to sterile 96-wells polystyrene microtiter plate (Orange Scientific, Braine-l’Alleud, Belgium) with 10 µL of the different phytochemicals at several concentrations (1, 3, 5, 8, 10, 13, 15, 18, 20 and 25 mM) and incubated at 37 °C for 1 h. Bacterial suspensions with DMSO (5%, v/v) and bacterial suspensions without phytochemicals were used as negative controls. Eugenol was used as positive control. Afterwards, 180 µL of the content of wells was removed and 180 µL of antimicrobial neutralizer composed by lecithin (3 g/L), polysorbate 80 (30 g/L), sodium thiosulfate (5 g/L), L-histidine (1 g/L), saponin (30 g/L) in phosphate buffer 0.25 mol/L at 1% (EN-1276, 1997) was added and allowed to act for 15 min. After that, 10 µL of each well was dropped on TSA plates. Finally, after 24 h of incubation at 37 °C, the plates were analyzed and the MBC of each phytochemical corresponded to the minimum concentration causing no growth on the TSA plates. The experiments were performed in triplicate and repeated three times.

### Biofilm formation and control using phytochemicals

Biofilm formation and control was performed according to Borges et al. (2017a). The cell density of the overnight grown bacteria was adjusted to approximately 10^7^ cells/mL in TSB. Then, 200 µL of the bacterial suspension were added to a 96-wells polystyrene microtiter plate and incubated at 37 °C during 24 h and under 160 rpm of agitation. After biofilm development, the medium was removed and the wells were washed twice with NaCl solution (8.5 g/L) in order to remove loosely attached bacteria. Then, 190 µL of NaCl solution (8.5 g/L) was added to each well with 10 µL of the phytochemicals at the MBC. Sessile bacteria with DMSO (5%, v/v) and sessile bacteria without phytochemical were used as negative controls. Eugenol was used as positive control. The microtiter plate was incubated at 37 °C and 160 rpm during 1 h. After that, the remaining attached bacteria were analyzed in terms of metabolic activity by the resazurin assay.

### Biofilm analysis by the resazurin assay

The metabolic activity of sessile bacteria was evaluated by the resazurin assay (Borges et al., 2014b; Ribeiro et al., 2017). This is a simple and non-reactive assay, where a non-fluorescent blue component is reduced by the living cells to a pink fluorescent component. After 1 h of incubation with the phytochemicals the content of the wells was removed and the wells were washed with NaCl solution (8.5 g/L). Then, 180 µL of fresh TSB was added to the wells. A volume of 20 µL of resazurin was added to each well (10%, Sigma-Aldrich, Portugal). Subsequently, the plate was incubated at 37 °C for 3 h and 160 rpm and the fluorescence intensity was measured in microplate reader (FLUOstar Omega, BMG Labtech, Ortenberg, Germany) at 530 nm excitation wavelength and 590 nm emission wavelength. Control experiments were performed on the growth inhibitory effects of DMSO and no inhibitory effects were found with DMSO at 5% (v/v) (available in the raw data file). The data reported were the average of four samples.

### Cytotoxicity of phytochemicals

Fibroblasts cell line L929 were cultured in alpha minimum essential medium (*α*-MEM; Gibco, Invitrogen, Carlsbad, CA, USA) supplemented with 10% (v/v) fetal bovine serum, 100 IU/mL penicillin, 100 µg/mL streptomycin and 2.5 µg/mL amphotericin B (all from Gibco, Invitrogen, Carlsbad, CA, USA), at 37 °C in a humidified atmosphere of 95% air and 5% CO_2_. At 70–80% confluence, the adherent cells were washed and detached with a trypsin solution (0.05% in 0.25% EDTA; both from Sigma-Aldrich, St. Louis, MO, USA) for 5 min at 37 °C. Cells were seeded on 48-well culture plates (Corning Incorporated, Corning, NY, USA) at a density of 3 × 10^4^ cells/cm^2^ and incubated for 24 h. Cells were then exposed to the different phytochemicals at MBC for 24 h. Afterwards, cell metabolic activity was evaluated using the resazurin assay (Ribeiro et al., 2017). Briefly, fresh complete medium containing 10% of resazurin (0.1 mg/mL; Sigma-Aldrich, St. Louis, MO, USA) was added to each condition and the plates were incubated for 3 h. The fluorescence intensity was then measured (530 nm excitation; 590 nm emission) using a microplate reader (FLUOstar Omega, BMG Labtech, Ortenberg, Germany). The data reported were the average of four samples. The results of the cell metabolic activity (MA) were expressed as percentage of the control group (DMSO; Sigma-Aldrich, St. Louis, MO, USA) by using the following Eq. (1):

(1) \begin{eqnarray*}MA(\text{%})=(MAp/MAc)\times 100\end{eqnarray*}

where *MAp* and *MAc* are the metabolic activity of the phytochemical and the control, respectively.

### Statistical analysis

The results were expressed as the average ± standard deviation. The statistical analysis of the results was done using the one-way analysis of variance (One-way ANOVA) followed by post hoc Tukey HSD multiple comparison test. Levels of *P* < 0.05 were considered to be statistically significant.

## Results

### Drug-likeness evaluation

**Figure 1:**
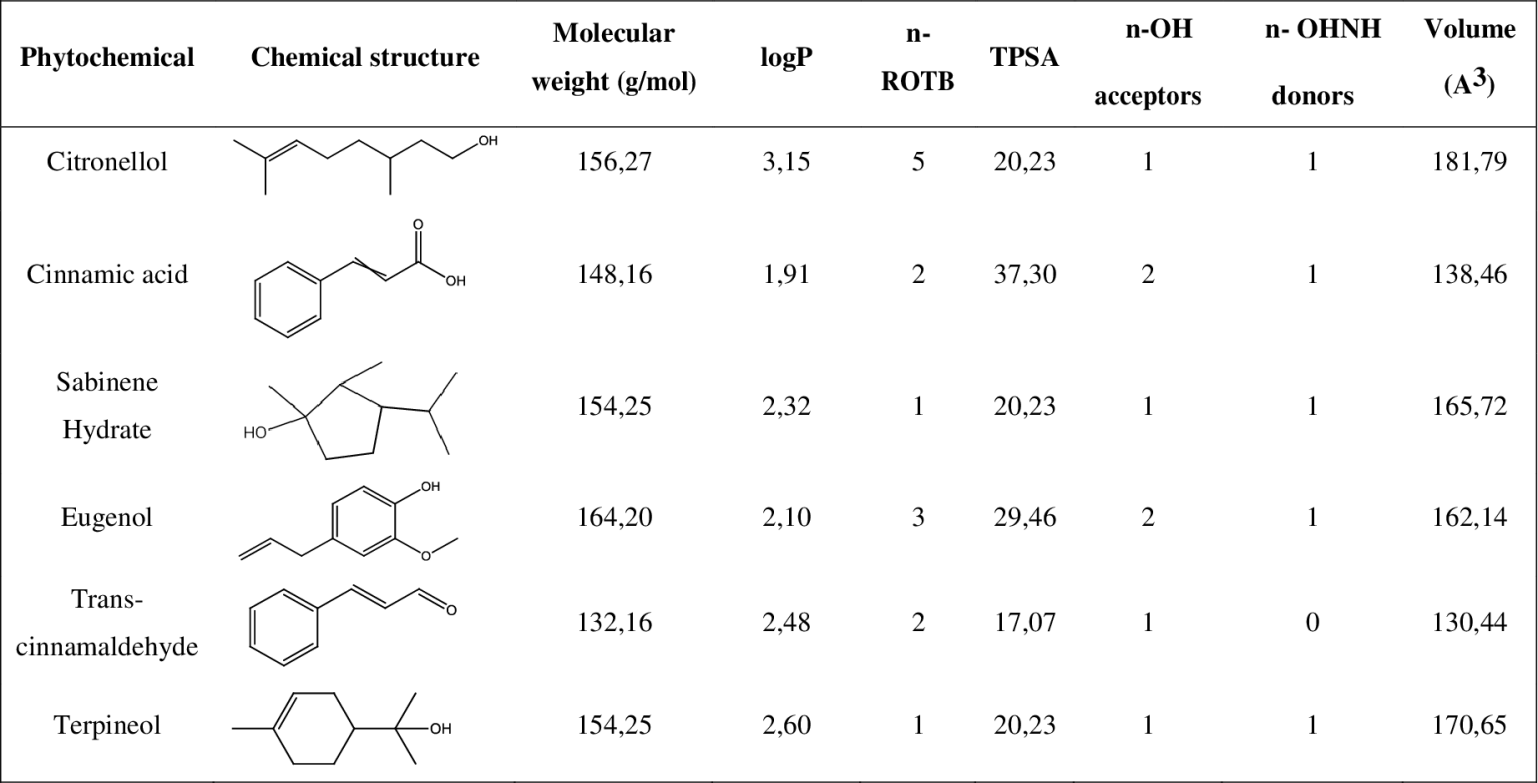
Chemical structure and molecular properties of selected phytochemicals. LogP, octanol-water partition coefficient; n-ROTB, number of rotable bonds; TPSA, topological polar surface area; n-OH, number of hydrogel bond acceptors; n-OHNH, number of hydrogen bond donors.

A drug-likeness evaluation was carried out focused on selected natural compounds and, for that, the chemical structure and molecular properties of the phytochemicals were assessed. As shown in Fig. 1, all the compounds presented an octanol-water partition coefficient (logP) ≤ 5, a molecular weight ≤ 500 Da (g/mol), a number of hydrogen bond acceptors ≤ 10 and a number of hydrogen bond donors ≤ 5. According to the Lipinski’s rule of five these are the requisites for the molecules to be considered as “drug-like compounds” (Lipinski, 2004; Lipinski et al., 2001). Additionally, all the phytochemicals presented a number of rotable bonds (n-ROTB) ≤ 5 and a topological polar surface area <40 Å.

### Antibacterial activity of phytochemicals on planktonic *S. mutans*

The antibacterial activity of selected phytochemicals was evaluated by MBC determination. As shown in Table 2, citronellol, cinnamic acid, sabinene hydrate, eugenol, trans-cinnamaldehyde and terpineol presented antibacterial activity against planktonic *S. mutans*. Moreover, the phytochemical that showed the lowest MBC was citronellol, being consequently the most effective.

**Table 2:** MBC values of the selected phytochemicals against *S. mutans*.

Phytochemical	MBC (mM)	MBC (µg/ml)
Citronellol	3	469
Cinnamic acid	5	741
Sabinene hydrate	10	1,542
Eugenol	10	1,642
Trans-cinnamaldehyde	13	1,728
Terpineol	15	2,314

### Antibacterial activity of phytochemicals on *S. mutans* biofilms

**Figure 2:**
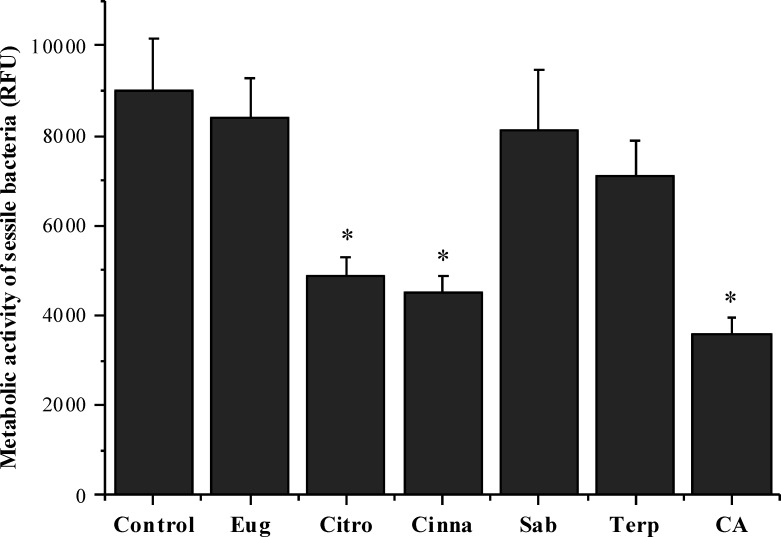
Antibacterial activity of different phytochemicals on *S.mutans* biofilm. *P < 0.05, significant reduction compared to control DMSO (5%, v/v) . Eugenol (Eug), citronellol (Citro), trans-cinnamaldehyde (Cinna), sabinene hydrate (Sab), terpineol (Terp), and cinnamic acid (CA).

The effects of the selected phytochemicals against pre-established 24 h-old *S. mutans* biofilms were evaluated in terms of metabolic activity. The selection of the suitable concentration of each compound for these anti-biofilm assays was based on the determination of its MBC against planktonic cells. As shown in Fig. 2, citronellol (46%), cinnamic acid (60%) and trans-cinnamaldehyde (50%) caused a statistically significant reduction of biofilm metabolic activity (*P* < 0.05). On the contrary, eugenol, sabinene hydrate and terpineol were not effective in inhibiting the biofilm (*P* > 0.05).

### Cytotoxicity of phytochemicals

**Figure 3:**
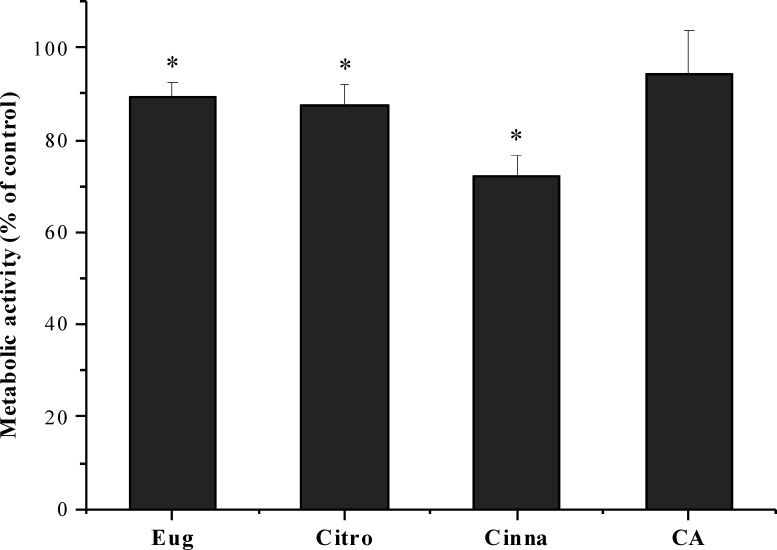
Influence of eugenol, citronellol, trans-cinnamaldehyde and cinnamic acid on fibroblast cells viability, as a percentage of cells on control. *P < 0.05, significant reduction compared to control DMSO (5%, v/v). Eugenol (Eug), citronellol (Citro), trans-cinnamaldehyde (Cinna), and cinnamic acid (CA).

The effect of citronellol, cinnamic acid and trans-cinnamaldehyde was evaluated on the fibroblast cell line L929. Eugenol was not effective in inhibiting *S. mutans* biofilms. However, it was also used as reference compound. The cells were exposed to the phytochemicals at MBC during a 24 h period. As shown in Fig. 3, the metabolic activity after exposure to phytochemicals was statistically lower than the control (5% DMSO, v/v), except for cinnamic acid, which did not present any statistically significant difference in cell viability (*P* > 0.05). Despite the slight decrease in metabolic activity for citronellol and eugenol, cell viability of 87 and 89% was obtained, respectively. The percentage of viable cells with trans-cinnamaldehyde was 72%, being the compound causing the most significant loss of cell viability.

## Discussion

The present work was undertaken to evaluate the antimicrobial potential of selected phytochemicals on both planktonic bacterial growth and biofilm formation of *S. mutans*, in order to search for new therapeutic antimicrobials to treat and prevent oral infectious diseases, particularly dental caries*.* Biofilm cells are known to be physiologically distinct from their planktonic counterparts, being surrounded by extracellular polymeric substances (EPS), which have a major role both in biofilm formation and maintenance through nutritive and protective functions. This peculiar form of biofilm development confers on the associated bacteria great resistance to conventional antimicrobial compounds (Davies, 2003; Del Pozo & Patel, 2007; Flemming & Wingender, 2010; Jagani, Chelikani & Kim, 2009; Nithya, Devi & Karutha Pandian, 2011). Therefore, the impact of antimicrobials on planktonic *S. mutans* cannot be compared to the effects on biofilm cells. In fact, biofilm resistance is multi-factorial and several mechanisms have been described, i.e., limited diffusion of antimicrobials through the biofilm matrix, enzyme-mediated resistance, distinct levels of metabolic activity inside the biofilm (from active to dormant state), genetic adaptation, efflux pumps and the presence of persister cells (Borges et al., 2016; Singh et al., 2017). It is clear that an accurate characterization of the antimicrobial action of a compound should focus cells in both planktonic and sessile states.

The drug-likeness of the selected compounds was evaluated using Lipinski’s rule of five (RO5) (Lipinski, 2004; Lipinski et al., 2001). This approach is based on several molecular properties. First, drug-like molecules should have a logP ≤ 5. It is equivalent to the ratio of concentrations of a compound in a mixture of octanol and water, two immiscible phases at equilibrium. The logP is used as a measure of hydrophobicity and therefore affects, among others, the drug bioavailability and mode of action. According to RO5, drug-like compounds should also have a molecular weight ≤ 500 g/mol to facilitate the intestinal and blood brain barrier permeability. Furthermore, the compounds should present a number of hydrogen bond acceptors ≤ 10 and a number of hydrogen bond donors ≤ 5. If a compound fails the RO5 there is a high probability that oral activity problems will be encountered as bad absorption or metabolism, for example (Lipinski, 2004; Lipinski et al., 2001). According to this rule, the selected phytochemicals assessed in this work have drug-like properties and, consequently, these compounds are potential drug leads.

The hydrophobic status of all the selected phytochemicals allows their interaction with the cell membrane of *S. mutans*, a Gram-positive bacterium. Contrary to Gram-negative bacteria, they lack an outer membrane but have a very tick cell wall, composed of approximately 90% of peptidoglycan and carbohydrates such as the teichoic acid (Tommasi et al., 2015). Moreover, the TPSA < 40 Å observed for all the compounds allow to conclude that they could have a good ability for penetrating cell membranes, as just the compounds with TPSA > 140 Å tend to have poor permeability (Veber et al., 2002). The most hydrophobic compounds are generally reported to be more toxic and the cytoplasmic membrane is often the primary site of antimicrobial action. Indeed, lipophilic compounds possess a high affinity for cell membranes by inducing changes in the membrane physicochemical properties. This effect is particularly reported for compounds with a logP > 3 (Ultee, Bennik & Moezelaar, 2002). In fact, citronellol (logP > 3) was found to be the most efficient antimicrobial phytochemical against planktonic *S. mutans*. Additionally, this compound was also effective on sessile bacteria at a concentration of just 3 mM. Previous studies have reported inhibitory effects of citronellol against biofilms of *Staphylococcus aureus* and *Escherichia coli* (Lopez-Romero et al., 2015). Another interesting finding of the present study was that this phytochemical presented higher antimicrobial activity than eugenol, a natural compound that has been widely used in dental care (Jadhav et al., 2004; Li et al., 2012; Xu et al., 2013). Citronellol is the only compound used in this study with a linear chemical structure, possessing a highly hydrophobic tail and a hydrophilic head, which makes it more prone to interact with the lipid bilayer of the cell membrane, disturbing the structures and rendering them more permeable (Bakkali et al., 2008). Citronellol is also the compound more flexible as its structure does not include any ring, and the n-ROTB of 5 confirms this higher molecular flexibility compared to all other compounds, which further helps to explain the higher antibacterial action.

Eugenol showed a lower antimicrobial effect than citronellol, with a MBC of 10 mM. This weakest antimicrobial activity could be attributed to its hydrophobicity being lower than 3 (logP = 2.10). Concerning the effects against sessile *S. mutans*, eugenol was not effective in causing inhibition. However, the antibiofilm potential of this compound has been reported by other authors against *Staphylococcus aureus* (Yadav et al., 2015), *Pseudomonas* species (Niu & Gilbert, 2004) and even against *S. mutans* (Xu et al., 2013). Nevertheless, the concentrations of eugenol tested by these authors against adhered *S. mutans* were higher than the concentration range used in this work. In a study performed by Malheiro et al. (2016) eugenol was also tested in a concentration of 10 mM against *S. aureus*, a Gram-positive bacterium, and no biofilm inhibition was observed.

Cinnamic acid, a phenolic acid, was the second compound with highest impact on *S. mutans*, presenting MBC of 5 mM. Furthermore, this compound also promoted a significant inactivation of sessile *S. mutans* at 5 mM. However, this phytochemical had a logP of 1.91 and was the compound with the smallest hydrophobicity. These results indicated that other factors than hydrophobicity must be involved. Phenolic acids are organic acids and their antimicrobial action is thought to be dependent on the concentration of undissociated acid. These small lipophilic molecules can cross the cell membrane by passive diffusion in their undissociated form, disturbing or even disrupting the cell membrane structure, acidifying the cytoplasm and causing protein denaturation (Malheiro et al., 2016). Malheiro et al. (2016) also observed that cinnamic acid exhibited significant antibiofilm activity against *S. aureus*.

Trans-cinnamaldehyde was also an effective compound against both planktonic and sessile *S. mutans*. This result corroborates previous studies that showed the significant inhibitory effect of trans-cinnamaldehyde against diverse bacterial pathogens (Mith et al., 2014; Sharma et al., 2016). These authors also observed that trans-cinnamaldehyde showed higher antibacterial activity than eugenol. A value of hydrophobicity lower than 3 (logP of 2.10) can help to explain the high MBC observed in this study.

The MBC found for the phytochemicals sabinene hydrate and terpineol was 10 mM and 15 mM, respectively. The lower antimicrobial activity against planktonic bacteria, for both compounds could be attributed to its hydrophobicity (logP < 3). Moreover, sabinene and terpineol were not effective in the control of sessile bacteria. Although, other authors observed the antibiofilm potential of these phytochemicals against *Escherichia coli* and *Staphylococcus aureus* (Borges et al., 2017a; Szweda & Kot, 2017). These results suggest that the antimicrobial efficacy of the natural compounds in controlling sessile bacteria appears to be dependent on the bacterial species.

In addition to the antibacterial action of these natural compounds, it is very important to understand their cytotoxicity before being used in humans. No obvious cytotoxic effects were detected for the phytochemicals eugenol, citronellol and cinnamic acid. Similarly, Babich & Visioli (2003) using 5 mM of cinnamic acid observed reduced effects on the viability of human gingival GN61 fibroblasts, human gingival S-G epithelial cells and human carcinoma HSG1 cells. In the present work, it was found that fibroblast cells were more sensitive to trans-cinnamaldehyde when compared to other phytochemicals with a cell viability of around 72%. Brari & Thakur (2015) also showed that cinnamaldehyde reduced viability of BV2 (microglia) cell line in a higher extent than citronellol and eugenol. According to ISO 10993-5 (2009), the differences observed were not significant in terms of toxicity, as cytotoxicity is considered when viability is lower than 70%. Therefore, these results showed that citronellol, cinnamic acid and trans-cinnamaldehyde presented antibacterial effects against planktonic and sessile *S. mutans*, without compromising the viability of fibroblasts cell line L929.

## Conclusions

Plant-derived molecules may offer a groundbreaking green approach to the discovery of broad-spectrum antimicrobials. The present work focused on the study of the antimicrobial effect of selected phytochemicals on planktonic bacterial growth and biofilm inhibition of *S. mutans*, as well as their toxic effects on a fibroblasts cell line. The phytochemicals citronellol, cinnamic acid and trans-cinnamaldehyde were the most effective in both inhibiting the growth of the planktonic *S. mutans* and causing significant biofilm inactivation. Moreover, these three compounds did not compromise fibroblast cell viability, suggesting that they may be new candidates for controlling oral infectious diseases. Data on the chemical properties of the selected phytochemicals propose that the molecular hydrophobicity seems to account for a higher antimicrobial effect.

##  Supplemental Information

10.7717/peerj.4872/supp-1 Data S1 Raw data

## Additional Information and Declarations

### Data Availability

The following information was supplied regarding data availability: Raw data can be found in Data S1.
